# Solid-Phase Collateral Cleavage System Based on CRISPR/Cas12 and Its Application toward Facile One-Pot Multiplex Double-Stranded DNA Detection

**DOI:** 10.1021/acs.bioconjchem.3c00294

**Published:** 2023-10-02

**Authors:** Hiroki Shigemori, Satoshi Fujita, Eiichi Tamiya, Shin-ichi Wakida, Hidenori Nagai

**Affiliations:** † Advanced Photonics and Biosensing Open Innovation Laboratory (PhotoBIO-OIL), National Institute of Advanced Industrial Science and Technology (AIST), Photonics Center Osaka University, 2-1 Yamada-Oka, Suita, Osaka 565-0871, Japan; ‡ Graduate School of Human Development and Environment, Kobe University, 3-11 Tsurukabuto, Nada-ku, Kobe, Hyogo 657-0011, Japan; § Institute of Scientific and Industrial Research (SANKEN), Osaka University, 8-1 Mihogaoka, Ibaraki, Osaka 567-0047, Japan

## Abstract

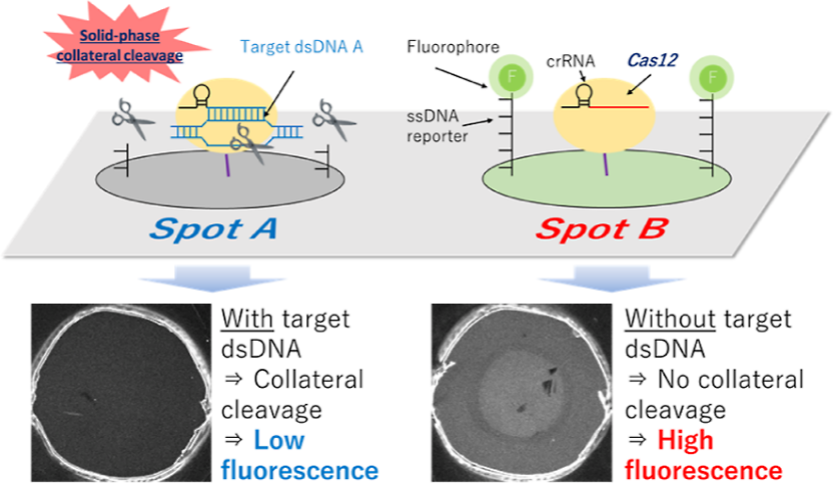

The clustered regularly interspaced short palindromic repeat (CRISPR)/CRISPR-associated protein 12 (Cas12) system is attracting interest for its potential as a next-generation nucleic acid detection tool. The system can recognize double-stranded DNA (dsDNA) based on Cas12-CRISPR RNA (crRNA) and induce signal transduction by collateral cleavage. This property is expected to simplify comprehensive genotyping. Here, we report a solid-phase collateral cleavage (SPCC) reaction by CRISPR/Cas12 and its application toward one-pot multiplex dsDNA detection with minimal operational steps. In the sensor, Cas12-crRNA and single-stranded DNA (ssDNA) are immobilized on the sensing surface and act as enzyme and reporter substrates, respectively. We also report a dual-target dsDNA sensor prepared by immobilizing Cas12-crRNA and a fluorophore-labeled ssDNA reporter on separate spots. When a spot captures a target dsDNA sequence, it cleaves the ssDNA reporter on the same spot and reduces its fluorescence by 42.1–57.3%. Crucially, spots targeting different sequences do not show a reduction in fluorescence, thus confirming the one-pot multiplex dsDNA detection by SPCC. Furthermore, the sequence specificity has a two-base resolution, and the detectable concentration for the target dsDNA is at least 10^–9^ M. In the future, the SPCC-based sensor array could achieve one-pot comprehensive genotyping by using an array spotter as a reagent-immobilizing method.

## Introduction

Currently, nucleic acid amplification testing (NAAT) is widely applied in many situations including infection diagnosis, cancer diagnosis, food analysis, and forensic science.^1^ Among NAAT technologies, real-time polymerase chain reaction (PCR) has been majorly adopted to identify target genes due to high sensitivity and specificity.^2^ However, real-time PCR is not straightforward to detect more than six target genes simultaneously in order to avoid the spectrum overlapping of individual fluorescent probes.^3^ To achieve comprehensive genotyping, DNA sequencing in the post-amplification process is used for accurate determination of both known and unknown genetic sequences.^4^ However, DNA sequencing has several limitations, including long running times (over 4 h).^5,6^ As an alternative, DNA microarray technology can be used to detect multiple nucleic acid amplicons using a wide variety of single-stranded DNAs (ssDNAs) immobilized on a sensing chip.^7^ Because ssDNA can be integrated using micro/nano-patterning, such as photolithography,^8^ array spotting,^9^ and inkjet-printing,^10,11^ DNA microarrays enable the simultaneous detection of approximately 300,000 targets on miniaturized chips in a single chamber.^12^ In addition, the amount of samples and reagents required for detection can be reduced.

Although some microarrays have been approved for use in infection diagnosis,^13,14^ they remain highly complex in terms of their operation. For example, many amplification technologies, such as PCR, recombinase polymerase amplification, and loop-mediated isothermal amplification (LAMP),^15^ generally produce double-stranded DNA (dsDNA). Hence, ssDNA synthesis (heat or alkali denaturation, secondary asymmetric PCR, and lambda exonuclease method)^16^ from amplicons is required to enable hybridization with the surface-immobilized ssDNA. In addition, a labeling process is required to convert the DNA hybrid into a signal and washing the unreacted target ssDNA and labeling reagents is also necessary.^17^ Therefore, multiplex DNA sensors with one or only a few operational steps are required to improve detection time, device miniaturization, and cost-effectiveness.

In recent years, the clustered regularly interspaced short palindromic repeat (CRISPR) and CRISPR-associated protein 9 (Cas9) system has drawn attention as an alternative nucleic acid sequence recognition tool for ssDNA–ssDNA hybridization. Considering that the Cas9 and CRISPR RNA (crRNA) complex can directly hybridize with target dsDNA via the complementary sequence to the crRNA,^18,19^ the application of CRISPR/Cas9 has been extended to not only gene editing but also nucleic acid detection.^20−22^ In previous reports, target dsDNA was captured by Cas9-crRNA immobilized on a sensor chip, and negative charge, change in electrical resistance, and the thickness of the captured dsDNA were detected by a graphene-based field effect transistor,^23,24^ a screen-printed electrode,^25^ and a biolayer interferometry detector,^26^ respectively. In addition, the colorimetric detection using Cas9-crRNA as an alternative to primary antibodies in enzyme-linked immunosorbent assays (ELISAs) has also been reported.^27^

In contrast, CRISPR/CRISPR-associated protein 12 (Cas12) is a more functional dsDNA detection tool than CRISPR/Cas9. Unlike the CRISPR/Cas9 system, Cas12-crRNA has a collateral cleavage activity that is induced by target dsDNA recognition and indiscriminately cleaves surrounding ssDNA.^28^ Therefore, CRISPR/Cas12 has attracted attention for use in signal transduction and amplification in dsDNA detection.^20−22,29^ Moreover, fluorescence detection after the cleavage of non-target ssDNA labeled with a fluorescent dye and quencher (F-Q reporter) is a representative method.^28,30−33^ For applications in resource-limited settings, electrochemical detection by a redox probe-labeled ssDNA reporter^34−36^ and colorimetric detection by a hapten-labeled ssDNA reporter and an immunochromatographic strip^31,37^ have also been reported. Although CRISPR/Cas12 achieves target recognition and signal transduction in one step, it is difficult to detect multiple targets in a “single pot.” The activated Cas12 cuts surrounding reporters regardless of their sequences; therefore, collateral cleavage-based multiplex detection generally requires the separation of CRISPR molecules into multiple liquid phases for each target of interest.^38^

To overcome this drawback, several approaches have been reported to avoid cross-collateral cleavage in multiplex detection. The first approaches are combinatorial arrayed reactions for multiplexed evaluation of nucleic acids (CARMEN)^39^ and microfluidic CARMEN^40^ technologies, which separate mixtures of samples and the CRISPR assay solution into a myriad of microwells. Although superior to other methods in terms of the number of microwells (9216 to 42,400 wells per chip), these methods have serious drawbacks, requiring multiple operating steps and instruments.

The second approach is exploitation of microfluidic technology to distribute sample solution into multiple assay areas with Cas12-crRNA and the ssDNA reporter.^41−46^ In contrast to the microwell-based separation of collateral cleavage reactions, in this process, users are simply required to apply the sample solution to the microfluidic chip inlet. However, increasing the assay area is difficult because long channels (centimeter order) between different assay areas are required to prevent cross-collateral cleavage. In addition, the amount of sample solution increases with an increase in assay area.

The third approach is the combination of Cas12 and some CRISPR-associated protein 13 (Cas13) subtypes to achieve one-pot multiplex detection by collateral cleavage.^31,47,48^ Taking into account that Cas13 targets RNA sequences and has a dinucleotide specificity to cleave non-target ssRNA, multiplex PCR-like detection can be realized by the classification of Cas types, reporter sequences, and fluorophore types for different targets. However, this dinucleotide specificity has been confirmed in only four subtypes of Cas13, and, thus, the ability to increase the number of detectable targets is limited. Furthermore, the Cas13-based detection of nucleic acid amplicons requires an RNA synthesis step.

Recently, the idea of Cas12-immobilized hydrogel microparticles (HMPs) has been introduced as a fourth approach.^49^ Considering that Cas12 is immobilized in individual HMPs, the sample, F-Q reporters, and myriad types of HMPs can be mixed in a single solution. However, the mixture must be loaded in a microfluidic chip to align and distinguish each HMP type. Afterward, the mixture in the microfluidic chip is replaced by oil to encapsulate the F-Q reporter in each HMP.

As mentioned above, many research groups have tried to achieve collateral cleavage-based multiplex detection of a single sample; however, to avoid signal-overlapping, the methods could not simultaneously achieve massively multiplexing and simple operation. Therefore, we hypothesize that the localization of all CRISPR molecules in the solid phase in an individual assay spot could enable one-pot comprehensive genotyping, unlike conventional methods in which all or some of the CRISPR molecules are diffused in the liquid phase. This phenomenon is similar to DNA microarrays, while operation steps are minimal due to the sequential reaction of dsDNA recognition and signal transduction by CRISPR/Cas12.

In this study, we immobilized both Cas12-crRNA and a fluorophore-labeled ssDNA reporter on the same sensing surface and studied the feasibility of the collateral cleavage reaction between them (solid-phase collateral cleavage: SPCC). Then, we applied the spot patterns immobilized with Cas12-crRNA and the ssDNA reporter for one-pot dual-target dsDNA detection. On the one hand, when the target dsDNA was present in the sample solution, the spots with complementary crRNA sequences showed a decreased fluorescence intensity. On the other hand, the spots that target other dsDNA sequences did not show decreased fluorescence intensities. Therefore, the developed dsDNA sensor based on the SPCC reaction can detect dsDNA sequences in a single reaction chamber with one-step recognition/signal transduction.

In the future, the SPCC-based sensing model is expected to enable the development of a comprehensive one-pot (over 100 targets) dsDNA sensor array by using an array spotter for the accumulation and immobilization of Cas12-crRNA and the ssDNA reporter.

## Results and Discussion

### Principle of the SPCC-Based Multiplex dsDNA Sensing Model

**Scheme 1:**
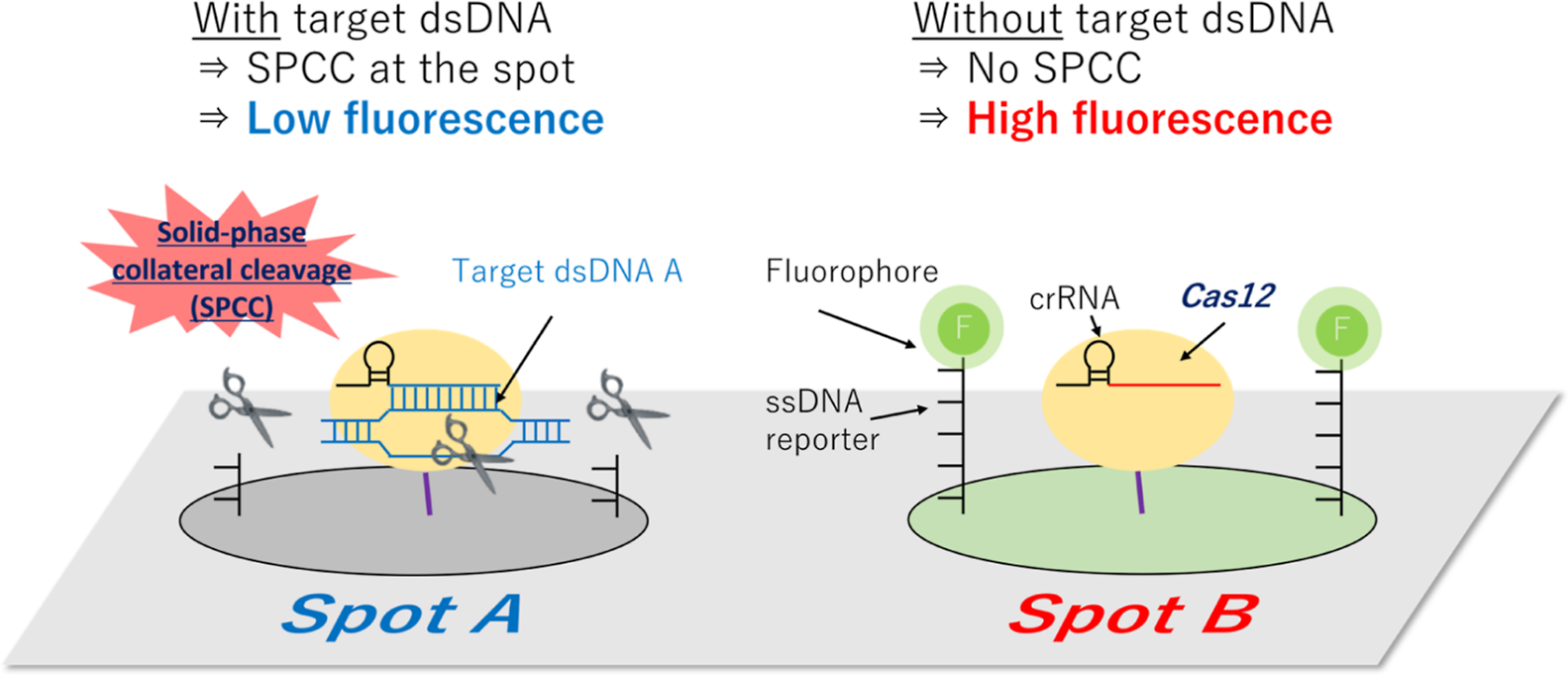
One-Pot Multiplex dsDNA Detection by the SPCC-Based Sensing Model dsDNA: double-stranded; SPCC: solid-phase collateral cleavage; Cas12: CRISPR-associated protein 12; crRNA: CRISPR RNA

Scheme 1 shows the detection principle of the SPCC-based multiplex dsDNA sensing model. Both Cas12-crRNA and the fluorophore-labeled ssDNA reporter are immobilized on the surface of each spot, and the crRNA sequence of each spot is set to be complementary to the target dsDNA sequence of each spot. When the sample is dropped on the sensor, the dsDNA in the sample is bound and captured on the spot, and its complementary crRNA sequence activates Cas12-crRNA. Then, the ssDNA reporter on the same spot is cleaved by the activated Cas12-crRNA, and the spot fluorescence intensity decreases. Given that the activated Cas12 is immobilized on the spot surface, it does not cleave the ssDNA reporter on other spots. Therefore, the target dsDNA sequence can be identified as the spot having decreased fluorescence.

### Collateral Cleavage of the Surface-Immobilized ssDNA Reporter and the Optimization of ssDNA Reporter Length

We studied whether the fluorescence-labeled ssDNA immobilized on the surface acts as the reporter for dsDNA via collateral cleavage from the activated Cas12 complex. First, to determine the concentration of ssDNA for deposition on the sensing surface, hexachlorofluorescein (HEX)-labeled ssDNA reporters (20, 40, 60, 80, and 100 nt) were amide-bonded to the carboxylic acid groups of 96-well ELISA plates. In this immobilization reaction, the concentration of the ssDNA reporter was optimized to 250 nM because the linear response of the surface fluorescence intensity with increasing ssDNA concentration reached 250 nM when using 20 nt ssDNA (Figure S1).

**Figure 1:**
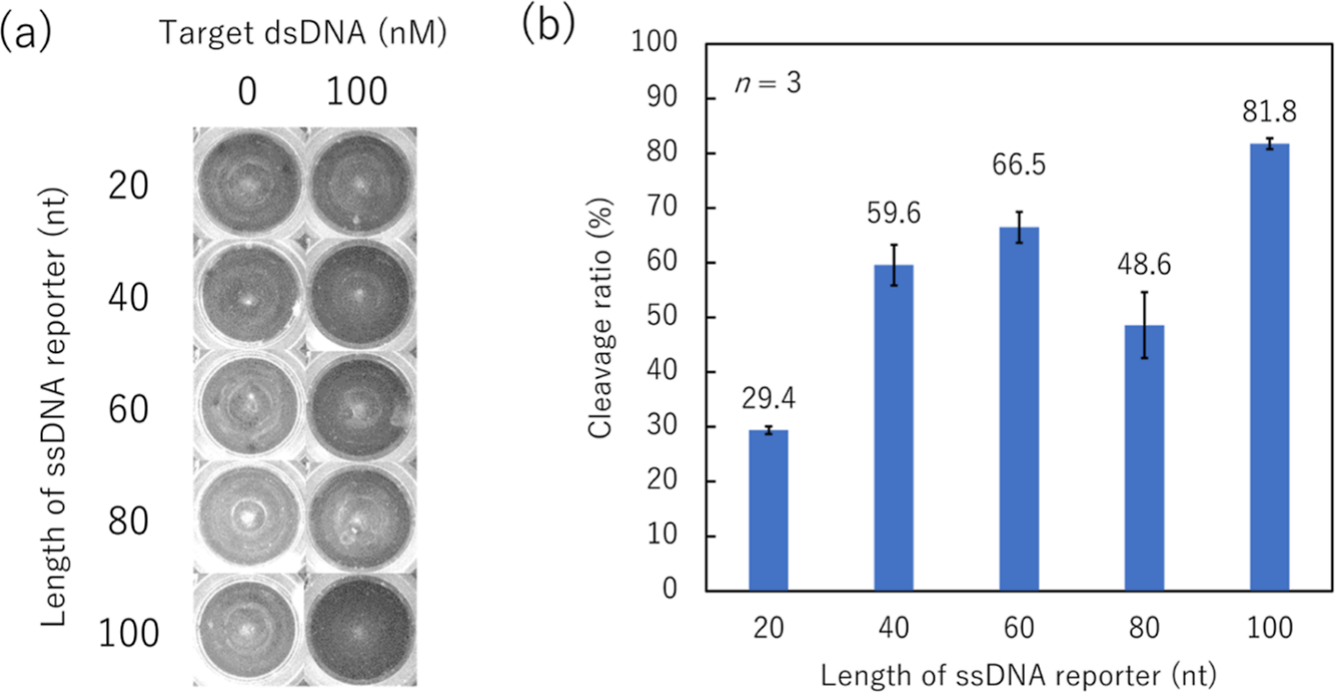
Collateral cleavage-induced reduction in the fluorescence of the ssDNA reporter (HEX-Poly T–NH_2_–20–100 nt)-immobilized surface. (a) Fluorescence images of each reporter length. (b) Cleavage ratio of the ssDNA. dsDNA: double-stranded; ssDNA: single-stranded.

After ssDNA immobilization, the solution including target dsDNA, Cas12, and crRNA was added dropwise into the respective wells. After washing, the fluorescence images of the respective wells were obtained (Figure 1a), and the fluorescence intensity was calculated as shown in Figure S2. There was variation in the fluorescence intensity of immobilized ssDNA without a target, which may be due to the efficiency of fluorophore-labeling or the efficiency of ssDNA immobilization. Nevertheless, all conditions showed decreased fluorescence in the presence of the target dsDNA. For precise evaluation, we calculated the cleavage ratio of ssDNA (the ratio in the fluorescence decrease from 0 nM dsDNA condition to 100 nM dsDNA condition) as shown in Figure 1b. The cleavage ratio of ssDNA tended to increase as the length of the ssDNA increased, except at 80 nt. This is because the number of cleavable sites increases as the length of ssDNA increases. On the other hand, the steric hindrance close to the well surface may prevent the access of Cas12 to the ssDNA where the ssDNA length was too short.

These results demonstrate that the surface-immobilized ssDNA can be used as the reporter material for collateral cleavage-based dsDNA detection, and the optimum ssDNA reporter length is 100 nt.

### Collateral Cleavage Activity of the Surface-Immobilized Cas12-crRNA and Optimization of Immobilization

**Figure 2:**
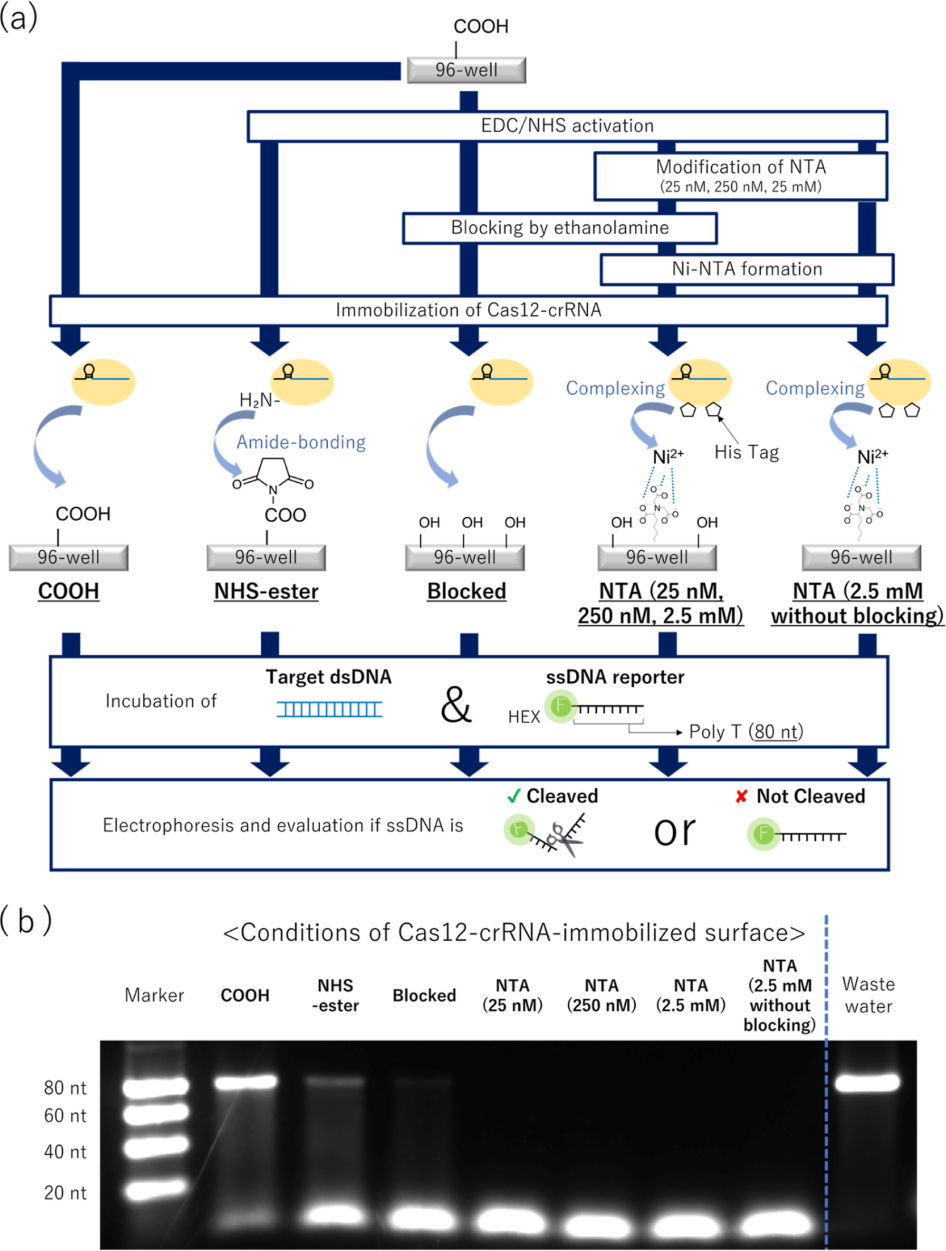
Collateral cleavage activity of the Cas12-crRNA-immobilized surface using agarose. (a) Schematic of Cas12-crRNA immobilization onto a 96-well plate and procedure to evaluate the collateral cleavage activity of the Cas12-crRNA-immobilized surface. (b) ssDNA reporter bands obtained from each Cas12-crRNA-immobilized surface. Cas12: CRISPR-associated protein 12; crRNA: CRISPR RNA; ssDNA single-stranded DNA; EDC: *N*-(3-dimethylaminopropyl)-*N*′-ethylcarbodiimide-hydrochloride; NHS: *N*-Hydroxysuccinimide; NTA: Nα,Nα-bis(carboxymethyl)-l-lysine hydrate.

Next, we optimized the surface immobilization of Cas12-crRNA by comparing two methods: the immobilization of the recombinant Cas12 protein with a C-terminal 6-His Tag through His Tag-Ni–NTA affinity (NTA: *N*_α_,*N*_α_-bis(carboxymethyl)-l-lysine hydrate) or covalent amide bonding. First, commercially available 96-well ELISA plates modified with carboxylic acid groups were prepared and modified according to some or all of the sequential reaction procedures shown in Figure 2a. Then, Cas12-crRNA was immobilized onto the modified surface (COOH, *N*-hydroxysuccinimide [NHS]-ester, blocked, and NTA: 25 nM, 250 nM, 2.5 mM, and 2.5 mM without blocking, respectively). To compare the collateral cleavage activity of each Cas12-crRNA-immobilized surface, the solution containing the target dsDNA and ssDNA reporter (80 nt) was dropped into the Cas12-crRNA-immobilized well. After incubation, the incubated solution was subjected to agarose gel electrophoresis (Figure 2a).

Figure 2b shows the bands of the ssDNA reporter. Bands from all surfaces appeared below 20 nt; the length of ssDNA before incubation was 80 nt. We also evaluated the cleavage activity of the last waste solution after the washing of unbound Cas12-crRNA, and a band only appeared at 80 nt. These results show that unbound Cas12-crRNA was completely washed out from the surface, and the Cas12-crRNA immobilized on the surface via specific/non-specific binding contributed to the cleavage of the ssDNA reporter.

However, uncleaved ssDNA reporter bands were observed for several surface conditions. Based on the brightness of the 80 nt bands, the cleavage activity of each surface decreased in the following order: NTA (25 nM and above) > blocked > NHS-ester > COOH. The Cas12-crRNA on the COOH surface was easily washed away as a result of weak bonding based on electrostatic interactions; therefore, the COOH surface is considered to have a low amount of Cas12-crRNA. Thus, the observation of the lowest cleavage activity for this surface is reasonable. Owing to the covalent bonding with the amino group of Cas12, the NHS-ester surface showed higher cleavage activity than the COOH surface, although it was lower than that of the blocked surface. The result is surprising but can be explained as the result of the molecular mobility of the surface-immobilized Cas12. Although an increased surface density of NHS-ester groups enhances the immobilization of Cas12 amino residues, an excessive surface density of NHS-ester groups could restrict the molecular mobility of Cas12 because of the increased number of multiple amide bonds per Cas12 molecule. Thus, the increased cleavage activity of the blocked surface may be caused by the high molecular mobility of the surface-immobilized Cas12 arising from the decrease in amide bonds. Among all surface conditions, NTA (25 nM and above) yielded the highest cleavage activity. Since Ni–NTA accesses only the 6-His Tag at the C-terminal of Cas12, the suitable orientation and high molecular mobility of the surface-immobilized Cas12 are thought to contribute to the activity. Furthermore, in contrast to the NHS-ester surface, Ni–NTA groups do not covalently bind to lysine residues near the dsDNA recognition region in Cas12.^50^ Therefore, the molecular recognition ability of Cas12 is thought to be maintained after Ni–NTA-based immobilization.

Overall, we confirmed that Cas12 has collateral cleavage activity, even after immobilization on a solid surface. Additionally, we identified the optimal immobilization conditions: NTA (25 nM and above), which yielded the highest cleavage activity. Although NTA (2.5 mM) and NTA (2.5 mM without blocking) showed no difference in cleavage activity, we determined that the blocking step is necessary because unblocked NHS-ester groups could lower Cas12 activity, as shown in the difference between the NHS-ester and blocked surface.

### SPCC-Based dsDNA Detection and Optimization

**Figure 3:**
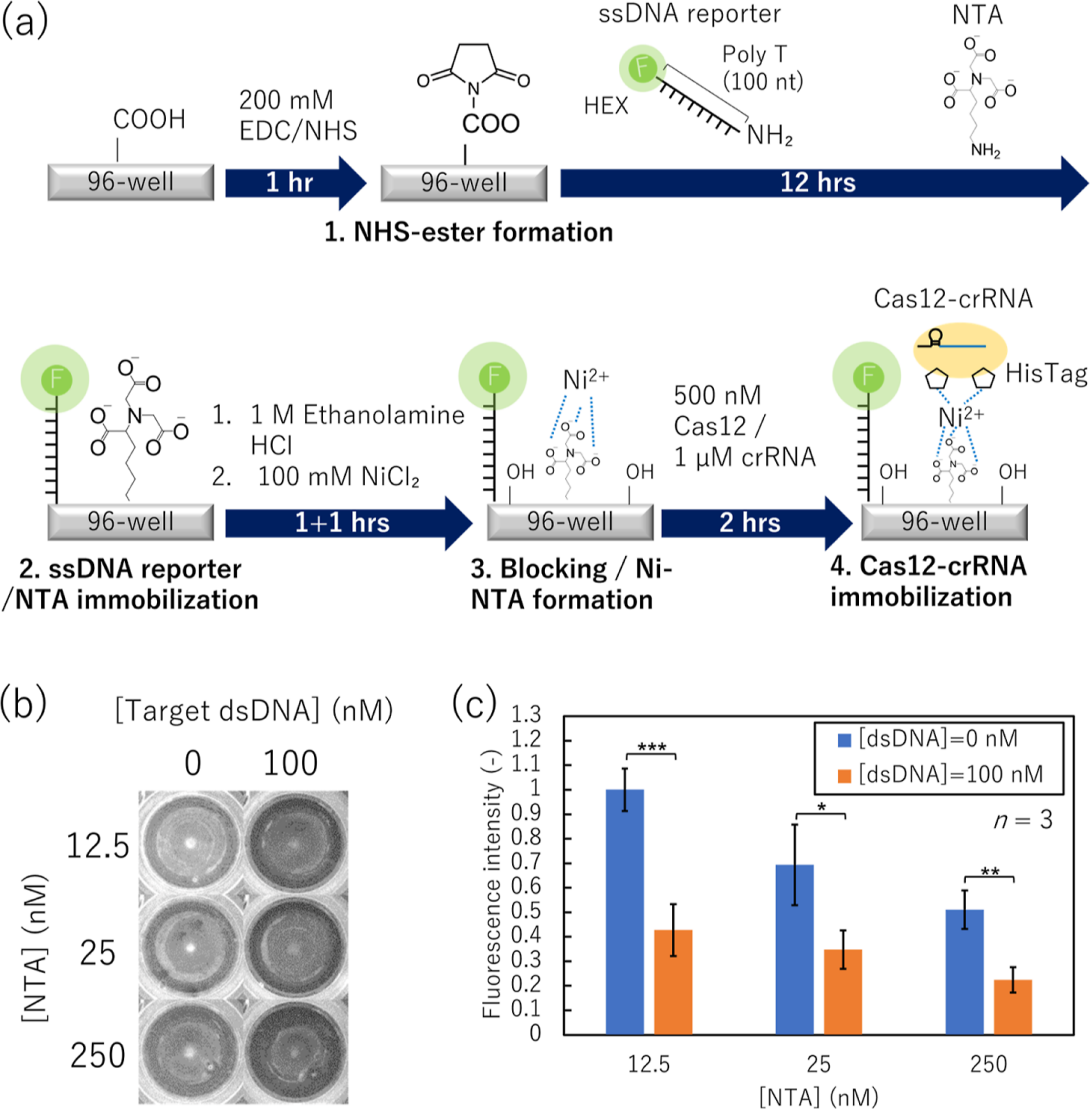
SPCC-based dsDNA detection on a 96-well plate immobilized with NTA of different concentrations. (a) Schematic of the immobilization of both Cas12-crRNA and the ssDNA reporter onto a 96-well plate. (b) Fluorescence images of samples having different NTA concentrations and (c) fluorescence intensity of the images (two-tailed Student’s *t*-test; *: *p* < 0.05, **: *p* < 0.01, ***: *p* < 0.005). SPCC: solid-phase collateral cleavage; dsDNA: double-stranded DNA; Cas12: CRISPR-associated protein 12; crRNA: CRISPR RNA; ssDNA: single-stranded DNA; EDC: *N*-(3-dimethylaminopropyl)-*N*′-ethylcarbodiimide-hydrochloride; NHS: *N*-Hydroxysuccinimide; NTA: *N*α,*N*α-bis(carboxymethyl)-l-lysine hydrate.

The Cas12-crRNA and ssDNA immobilized on the sensing surface were found to act as enzyme and reporter substrates, respectively, in the collateral cleavage reaction. Hence, we attempted to immobilize both Cas12-crRNA and ssDNA on the same surface to test and optimize the SPCC-based dsDNA detection. Thus, we fabricated a sensing surface immobilized with both Cas12-crRNA and the ssDNA reporter following the procedure shown in Figure 3a.

When we simultaneously immobilized both NTA and the ssDNA reporter, the concentrations of NTA were 12.5, 25, and 250 nM. The concentration of the ssDNA reporter was constant at 250 nM; therefore, the NTA/ssDNA reporter concentration ratios were 1:20, 1:10, and 1:1, respectively. After the incubation of 0 or 100 nM target dsDNA, fluorescence images of each surface were obtained after washing (Figure 3b), and the fluorescence intensity was calculated as shown in Figure 3c. In addition, the Δ*F* (the difference in the fluorescence intensities of the 0 and 100 nM dsDNA samples) values and surface cleavage ratios are shown in Figure S3a,b, respectively.

When target dsDNA was not applied to the surface, the fluorescence intensity decreased with an increase in NTA concentration because of the decrease in the concentration of the ssDNA reporter per quantity of NTA. Although the fluorescence intensity without target dsDNA varied for each NTA concentration, the fluorescence was significantly decreased with 100 nM target dsDNA in every case. Therefore, the SPCC system shows potential for dsDNA detection. Although the Δ*F* value increased as the NTA concentration decreased (Figure S3a), the cleavage ratio remained at 50–60% regardless of the NTA concentration (Figure S3b). Thus, the sufficient [NTA] was found to be at least 12.5 nM; hence, this was determined to be the optimum value.

### One-Pot Dual-Target dsDNA Detection Based on the SPCC Reaction and Its Analytical Performances

To confirm SPCC-based one-pot dual-target dsDNA detection without cross-cleavage, we patterned the Cas12-crRNA/ssDNA reporter-immobilized surface to produce a dual-target dsDNA sensor (Figure S4). Before the immobilization of the CRISPR reagents, plate seals were cut into circle shapes with two holes and fixed to the bottom surfaces of the wells. Afterward, 2.5 μL of each reagent was dropped onto each spot in the order shown in Figure 3a.

**Figure 4:**
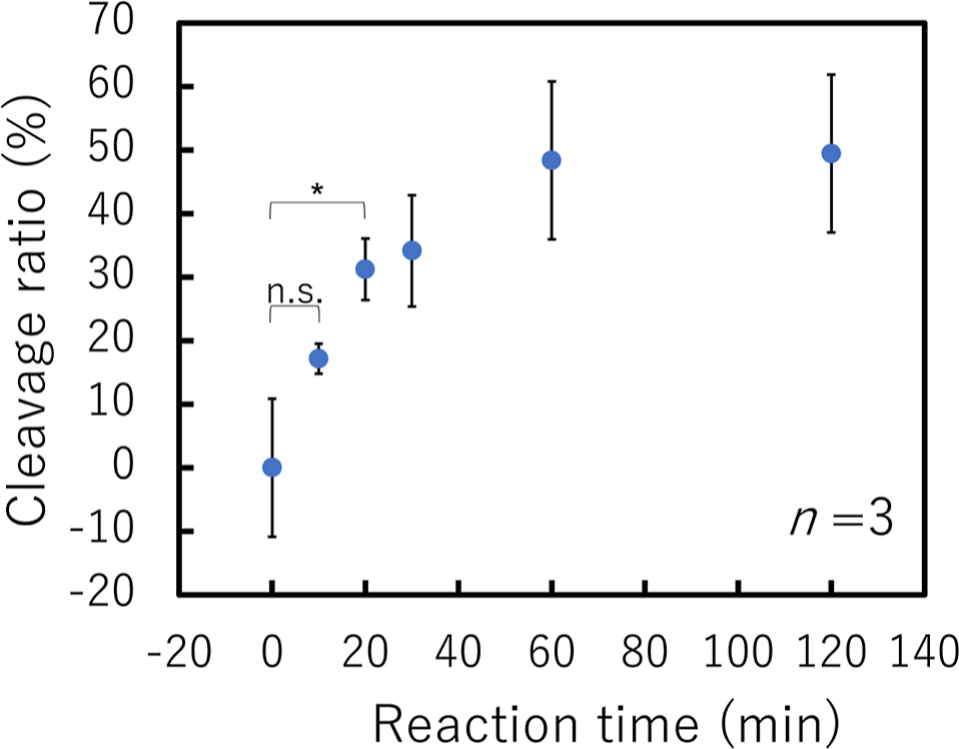
Relationship between the cleavage ratio of the Cas12-crRNA/ssDNA reporter-immobilized surface and reaction time with 100 nM pVenus-N1 (two-tailed Student’s *t*-test; n.s.: not significant, *: *p* < 0.05). Cas12: CRISPR-associated protein 12; crRNA: CRISPR RNA; ssDNA: single-stranded DNA.

First, we optimized the reaction time of the collateral cleavage reaction on the developed sensing spot. After incubation of the 100 nM target dsDNA and washing, the cleavage ratio of the patterned spot increased, as shown in Figure 4. The collateral cleavage-induced fluorescence decrease continued for 60 min; thus, a reaction period of 60 min or more was selected to study the analytical performance. In experimental results described later, the incubation time was set to 120 min for the entire completion of the SPCC. Further, the Student’s t-test analysis of the cleavage ratio at 0 min and those at later times revealed that 100 nM target dsDNA could be detected after 20 min. Considering that current nucleic acid amplification technologies (amplification capacity: approximately 10^11^) can easily produce 100 nM dsDNA from as little as 1 copy/μL template,^15^ this detection time indicates the potential of these sensing spots for use in speedy genetic diagnosis by integration with rapid nucleic acid amplification systems such as microfluidic PCR^51^ and LAMP.^52^

Next, we performed dual-target dsDNA detection on the sensing spots and evaluated the sequence specificity of each spot. We selected two types of PCR amplicons from pEGFP-N1 and pVenus-N1 plasmids as target dsDNAs and deposited the pEGFP-N1-targeting crRNA and pVenus-N1-targeting crRNA onto individual patterned spots. As shown in the sequence list (Table S1), two types of 17 nt-hybridization ranges have two-base differences. We tested the ability of the sensor to simultaneously detect both pairs by applying 80 μL of the sample solution to two spots on a surface.

**Figure 5:**
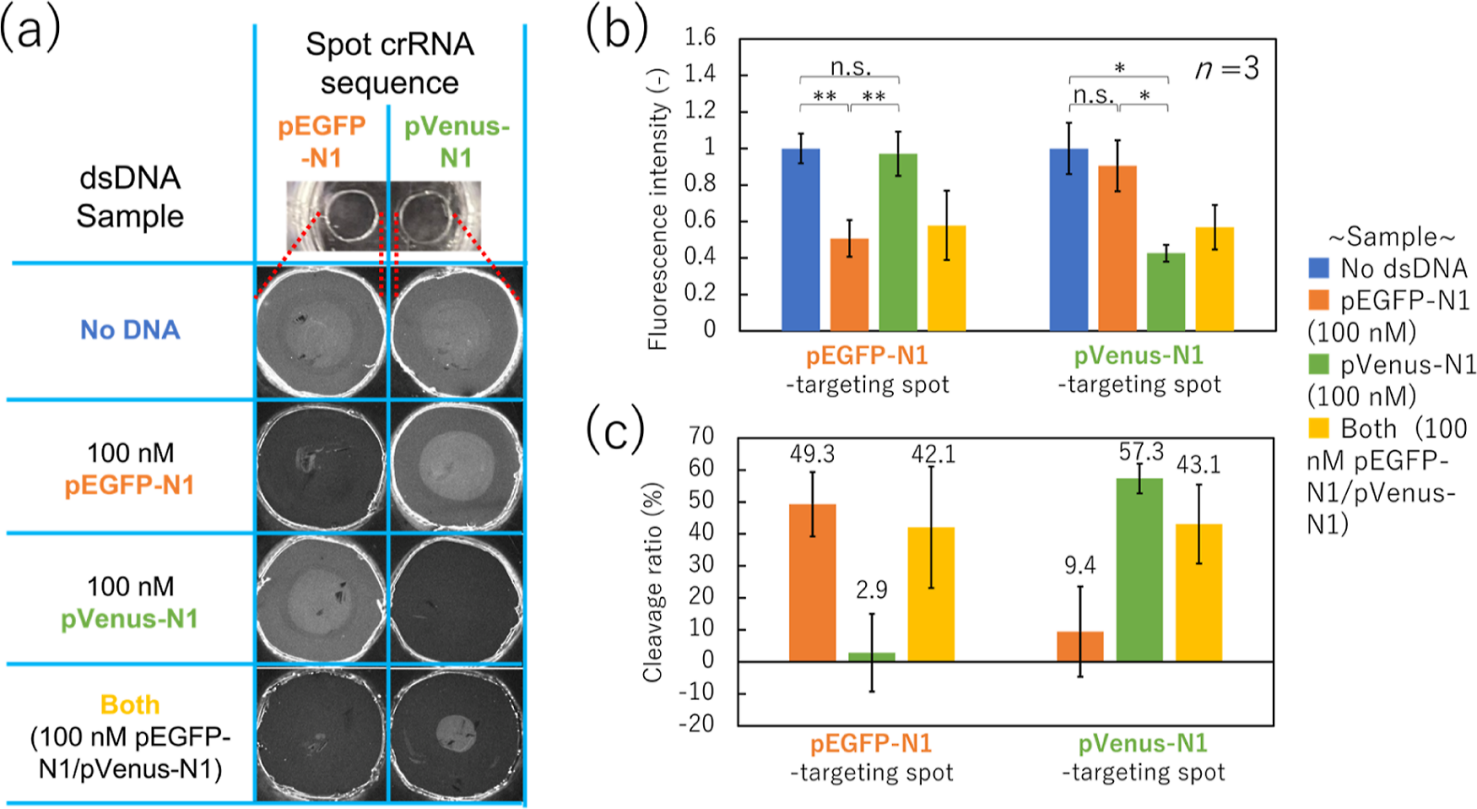
One-pot dual-target dsDNA detection using the SPCC-based sensor. (a) Fluorescence images of each spot after sample incubation. (b) Fluorescence intensities (two-tailed Student’s *t*-test; n.s.: not significant, *: *p* < 0.05, **: *p* < 0.01) and (c) cleavage ratio of each spot. SPCC: solid-phase collateral cleavage; dsDNA: double-stranded DNA.

Figure 5a shows the fluorescence images of each spot after incubation of 0 or 100 nM pEGFP-N1 and pVenus-N1 target dsDNAs and washing. Their fluorescence intensities and cleavage ratios are shown in Figure 5b,c, respectively. When the target dsDNA was included in the incubated sample, pEGFP-N1-targeting and pVenus-N1-targeting spots yielded cleavage ratios of 49.3 and 57.3%, respectively. In contrast, the spot fluorescence intensity in response to dsDNA having a two-base difference was not significantly different from that in response to the sample without any dsDNA. Therefore, the SPCC-based sensor can detect dual-target dsDNA sequences in a single chamber without interference between spots. In addition, the sequence specificity was found to have at least a two-base resolution. To clarify the strong points of our SPCC reaction scheme, we summarize the properties of the conventional liquid-phase collateral cleavage reaction for multiplex nucleic acid detection methods that are mentioned in the introduction section. As shown in Table S2, the spots per chip size (= the potential for the accumulation of assay spots) of our SPCC-based sensor (5.88 spots/cm^2^) is higher than that of a microfluidic-based multi-chamber (≤2.18 spots/cm^2^). According to instruments and operation steps, our platform is operated by a fluorescence microscope with three-step operations (sample injection, washing, and fluorescence detection) whereas microwell and HMP-based multi-chamber methods require several instruments and four or more steps. Owing to the multiplex dsDNA detection in a single chamber, the SPCC-based sensing model is expected to be a useful tool to increase the number of detectable targets and to simplify instruments and operation steps.

Focusing on the cleavage ratio with the target sequence, the pEGFP-N1-targeting spot produced a slightly lower cleavage ratio than the pVenus-N1-targeting spot. As shown by the values in Table S1, there is a complementary sequence pair (5′-UGAA-3′ and 5′-UUCA-3′) in the pEGFP-N1-targeting crRNA sequence; thus, there is a risk of intramolecular hybridization. Therefore, we hypothesized that the intramolecular hybridization reduced the amount of crRNA introduced into Cas12 and consequently decreased the reactivity of the pEGFP-N1-targeting spot. Based on this hypothesis, it is important to avoid complementary sequence pairs in the crRNA design.

When the target dsDNAs encoding the pEGFP-N1 and pVenus-N1 sequences were mixed and dropped on two spots, the fluorescence intensities of both the pEGFP-N1-targeting and pVenus-N1-targeting spots decreased to the same level as in the single-target condition. Therefore, our SPCC-based sensing model is expected to have applications in the analysis of systems containing multiple targets. For example, in the detection of multiple mutations and co-infections for infection diagnosis, meat species identification tests, and environmental DNA surveys. The reason the copresence of pEGFP-N1 and pVenus-N1 target dsDNA resulted in slightly lower cleavage ratios than the single-target conditions is likely the competitive recognition of the two targets. In Cas12-based dsDNA recognition, crRNA hybridizes to target dsDNA sequences while unwinding the hybrid formed from the protospacer adjacent motif (PAM) region (5′-TTTN-3′) in the 5′ to 3′ direction.^53,54^ Thus, if the crRNA encountered mismatch points during hybridization in the 5′ to 3′ direction, Cas12 would not be activated^54^ but rather unwind the hybrid.^53^ Given that the pEGFP-N1 and pVenus-N1 target dsDNA sequences share a five-base common sequence (5′-TGAAG-3′) between the PAM and the mutation site, the competitive recognition of the common sequence is thought to occur and result in a decreased cleavage ratio. To further improve the cleavage ratio, crRNA should be designed in such a way that the distance between the PAM and the mutation site is as short as possible.

Since the PAM sequence in target dsDNA is necessary to be recognized by Cas12, it can be easily inserted into any dsDNA sequences by using a PAM-fused primer during PCR, as shown in the experimental procedures and Table S1. Other approaches to remove the PAM limitation include the use of AaCas12b, which sets short PAM (5′-TTN-3′)^55^ and the assay reaction with T_m_-reducing reagent at 48 °C.^56^

**Figure 6:**
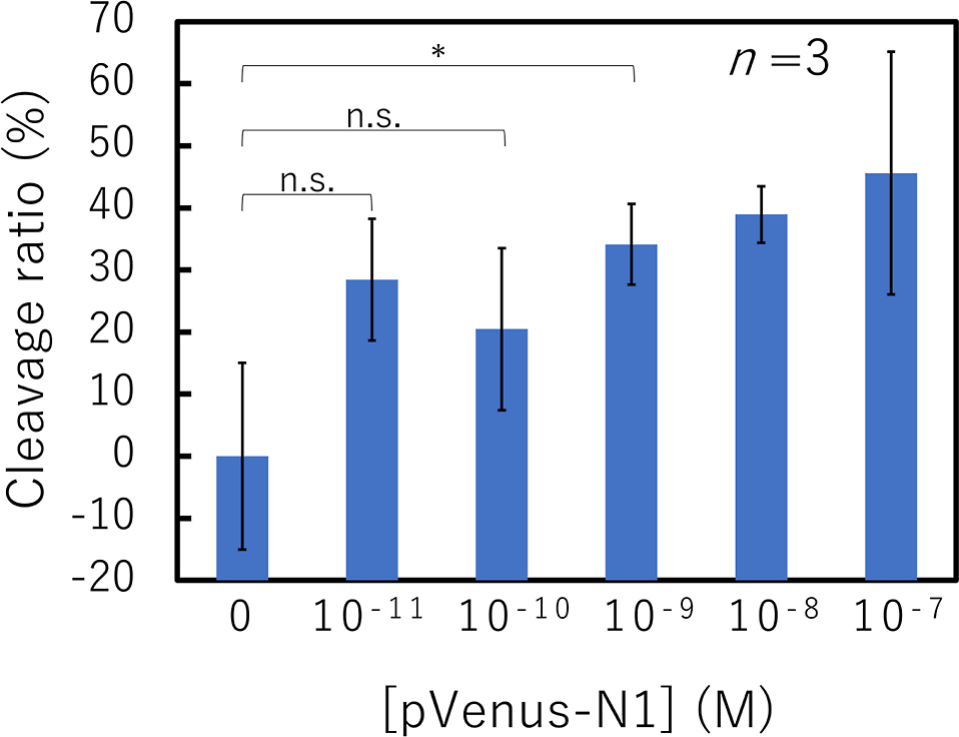
Relationship between the cleavage ratio of the pVenus-N1-targeting spot and the pVenus-N1 concentration (two-tailed Student’s *t*-test; n.s.: not significant, *: *p* < 0.05).

Finally, we evaluated the sensitivity of the Cas12-crRNA/ssDNA reporter-immobilized spot. Figure 6 shows the cleavage ratios of the pVenus-N1-targeting spot in the presence of various concentrations of pVenus-N1 after 2 h of incubation and washing. The cleavage ratio increased with an increase in the pVenus-N1 concentration. Analysis using Student’s t-test revealed that the cleavage ratios of the spots with 10^–9^ M or greater pVenus-N1 were significantly higher than that of the 0 M condition. Therefore, the detectable concentration of the Cas12-crRNA/ssDNA reporter-immobilized spot was found to be at least 10^–9^ M. Since the diffusion of Cas12 and the ssDNA reporter is restricted to achieve one-pot multiplex detection, it is natural to have lower sensitivity than liquid-phase CRISPR/Cas12-based single-plex detection (femto-molar to sub-nano-molar order sensitivity).^57^ However, DNA microarray technology is commercially available despite its sensitivity being equivalent to that of SPCC-based sensing models.^58−60^ Therefore, the idea of prioritizing multiplexing over sensitivity is widely accepted as a developmental policy for nucleic acid sensors in the post-amplification process. Additionally, because Cas12 directly hybridizes to dsDNA and the fluorescent probe is pre-deposited, our SPCC-based sensing model can detect target dsDNA by only the hybridization and washing steps. In contrast, DNA microarray technology requires additional ssDNA synthesis and labeling steps (Figure S5). Therefore, we conclude that our SPCC sensing model is sensitive enough to identify nucleic acid amplificons as well as simplify the detection process.

## Conclusions

In this study, we developed an SPCC-based sensing model and applied it in one-pot dual-target dsDNA detection. The long ssDNA reporter was found to be easily cleaved by Cas12. In addition, Cas12 immobilized by His Tag-Ni–NTA showed high collateral cleavage activity. The fabricated SPCC-based sensor successfully identified two target dsDNA sequences with two-base differences in a single chamber under the optimized reaction conditions. Furthermore, the detectable concentration for the target dsDNA was approximately 10^–9^ M.

Although there were only two detectable targets in this study, the new insight that surface immobilization of CRISPR molecules is useful to avoid cross-collateral cleavage contributes to the integration of assay spots in CRISPR-based multiplex diagnostics. We believe that spot dispensing by an array spotter or an inkjet printer would enable the number of detectable targets to be increased to the same level as those of conventional DNA microarrays and collateral cleavage in individual microwells.

Regarding the operating procedure of our prototype, a washing step is still required (as for conventional DNA microarrays and Cas9-crRNA-immobilized sensors). However, the immobilization of the F-Q ssDNA reporter or the use of an evanescent microscope as a detector could enable one-step detection. We also expect that the extension of the linker length using materials such as polyethylene glycol, peptide, and oligonucleotides (without ssDNA) may contribute to a further increase in the cleavage ratio. Furthermore, the ssDNA reporter could be labeled with methylene blue or ferrocene, thereby allowing for SPCC-based multiplex detection to be performed on a microelectrode array, which is lower cost and smaller than a fluorescence detector.

Overall, we believe that our SPCC is an ideal tool for comprehensive yet simple genotyping and has a wide range of biosensing applications, such as infection diagnosis, cancer cell identification, meat inspection, and environmental DNA surveying.

## Experimental Procedures

### Materials and Reagents

Alt-R L.b. Cas12a (Cpf1) Ultra was purchased from Integrated DNA Technologies, Inc. (Coralville, IA, USA). CRISPR RNA (crRNA) was purchased from Thermo Fisher Scientific Inc. (Waltham, MA, USA). pEGFP-N1 plasmid was obtained from Clontech Laboratories, Inc. (Mountain View, CA, USA), and pVenus-N1 plasmid was synthesized by inducing point mutations in the pEGFP-N1 sequence. PCR primers and the ssDNA reporter were purchased from Eurofins Genomics K.K. (Tokyo, Japan). All oligonucleotide sequences are listed in Table S1. *N*-Hydroxysuccinimide (NHS), *N*-(3-dimethylaminopropyl)-*N*′-ethylcarbodiimide-hydrochloride (EDC), *N*_α_,*N*_α_-bis(carboxymethyl)-l-lysine hydrate (NTA), and ethanolamine hydrochloride were purchased from Sigma-Aldrich Co. LLC (St. Louis, MO, USA). 2-(*N*-Morpholino)ethanesulfonic acid (MES) was purchased from Dojindo Molecular Technologies, Inc. (Kumamoto, Japan). Sodium hydroxide, sodium dihydrogenphosphate dihydrate, disodium hydrogen phosphate dodecahydrate, and nickel(II)chloride were purchased from Fujifilm Wako Pure Chemical Corporation (Osaka, Japan). Hy Agarose was purchased from HydraGene Co., Ltd. (Xiamen, China). Ambion Nuclease-Free Water was purchased from Thermo Fisher Scientific Inc. (Waltham, MA, USA). SpeedSTAR HS DNA polymerase, an RNase inhibitor, and 6× loading buffer were purchased from Takara Bio Inc. (Kusatsu, Japan). A QIAquick PCR purification kit was purchased from QIAGEN (Venlo, The Netherlands). Further, 10× NEBuffer 3 was purchased from New England BioLabs (Ipswich, MA, USA). TBE Buffer (10×) was purchased from SERVA Electrophoresis GmbH (Heidelberg, Germany). Carboxyl-type 96-well ELISA plates (#MS-8708F) were purchased from Sumitomo Bakelite Co., Ltd. (Tokyo, Japan). Microplate Sealing Tape Polyolefin (#9795) was purchased from 3M (Saint Paul, MN, USA).

### Instrumentation

A Takara Dice Touch thermal cycler (Takara Bio Inc., Kusatsu, Japan) was used for the amplification of the target dsDNA and the denaturation of the crRNA. A NanoDrop One microvolume spectrophotometer was used to measure the concentration of the target dsDNA. A SANYO MOV-112 (U) drying oven (SANYO Electric Co., Ltd., Osaka, Japan) was used to control the temperature of the collateral cleavage reaction. An OLYMPUS MVX10 macro zoom fluorescence microscope system (Olympus Corporation, Tokyo, Japan), OLYMPUS U-HGLGPS light source (Olympus Corporation, Tokyo, Japan), and ImagEM EM-CCD camera (Hamamatsu Photonics K.K., Hamamatsu, Japan) were used to take a fluorescence image of an ELISA plate surface. A Mupid-exU horizontal electrophoresis apparatus (Takara Bio Inc., Kusatsu, Japan) and a ChemDoc Imaging System (Bio-Rad Laboratories, Inc., Hercules, CA, USA) were used for the agarose gel electrophoresis of the ssDNA reporter and for imaging the bands, respectively. A Silhouette Cameo 4 auto-cutter (Silhouette, Lindon, UT, USA) was used for cutting the microplate seal.

#### Preparation of Target dsDNA

First, pEGFP-N1 and pVenus-N1 dsDNAs were amplified, and the PAM sequence (5′-TTTN-3′) was inserted into the amplified dsDNA sequence by PCR. The components of the PCR mixture are listed in Table S3, and the thermal cycling procedure was (1) 98 °C for 30 s, (2) 98 °C for 10 s, (3) 59 °C for 10 s, (4) 72 °C for 10 s [repeat (2)–(4) 40 times], (5) 72 °C for 2 min, and (6) 4 °C hold. Subsequently, the amplified dsDNA was purified using a QIAquick PCR purification kit according to the manufacturer’s instructions.

#### Immobilization of ssDNA on a 96-Well ELISA Plate and the Collateral Cleavage Reaction of Immobilized ssDNA

First, the carboxylic acid groups of the 96-well-plate surface were activated by incubation with 200 mM EDC/NHS in 100 μL of 50 mM MES-NaOH buffer (pH 6.0) for 1 h. Then, each well was rinsed three times with 100 μL of water. After the activation with NHS-ester groups, 250 nM ssDNA reporter in 100 μL of 100 mM phosphate buffer (pH 7.5) was dropped on each well and incubated for 12 h, and the well was rinsed five times with 100 μL of water.

The collateral cleavage reaction was performed by dropping 60 μL of cleavage solution into the ssDNA-immobilized well (the components of the cleavage solution are listed in Table S4). After incubation for 1 h, each well was rinsed five times with 100 μL of water. Finally, fluorescence images (λ_ex_ = 535–555 nm, λ_em_ = 570–625 nm) of the well were obtained by microscopy and analyzed using Image J (NIH, Bethesda, MD, USA). The fluorescence intensity was calculated as the increase in the ratio of the analyzed intensity relative to the background (that is, a well without the reporter).

#### Immobilization of the Cas12-crRNA Complex on a 96-Well ELISA Plate and Collateral Cleavage Activity by Agarose Gel Electrophoresis

Cas12-crRNA complex was immobilized on a 96-well ELISA plate as shown in Figure 2a. NHS-ester activation was performed by dropping 200 mM EDC/NHS in 100 μL of 50 mM MES-NaOH buffer (pH 6.0) into 96-well ELISA plates modified with carboxylic acid groups. After incubation for 1 h, each well was rinsed three times with 100 μL of water. NTA immobilization was performed using 0–25 mM NTA in 100 μL of 100 mM phosphate buffer (pH 7.5), which was dropped into the wells and incubated for 12 h. Subsequently, each well was rinsed with 100 μL of water. Blocking was performed by adding 1 M ethanolamine hydrochloride in 100 μL of 100 mM phosphate buffer (pH 7.5), followed by incubation for 1 h. Then, the well was rinsed with 100 μL of water. Ni–NTA complex formation was performed by adding 100 μL of 100 mM nickel(II)chloride into each well, followed by incubation for 1 h. Then, each well was rinsed with 100 μL of water. Finally, 500 nM Cas12 and 1 μM crRNA (targeting pVenus-N1) in 60 μL of 1× NEBuffer 3 were dropped onto each surface and incubated for 2 h, and each well was rinsed five times with 100 μL of water.

After the functionalization of each surface, the collateral cleavage activities were evaluated. First, 100 nM pVenus-N1 amplicon and 500 nM ssDNA reporter (HEX-Poly T–NH_2_–80 nt) were dissolved in 1x NEBuffer 3 with 0.5 U/μL RNase inhibitor. Then, 60 μL of the solution was added dropwise into each prepared well and incubated for 2 h. Finally, 10 μL of the incubated solution was mixed with 2 μL of 6× loading buffer and applied to the agarose gel for electrophoresis (gel concentration: 3 wt %, buffer: TBE, voltage: 100 V, migration time: 30 min). Band images were obtained using a ChemDoc Imaging System.

To evaluate if unbound Cas12 was left on the surface, 15 μL of rinse water after the fifth rinse of the Cas12-crRNA-immobilized surface was mixed with 5 μL of 4-fold-concentrated target dsDNA and ssDNA reporter solution. After incubation at 37 °C for 2 h, the incubated solution was applied for agarose gel electrophoresis, and band images were obtained using the aforementioned procedure.

#### Simultaneous Immobilization Process of Both Cas12 and the ssDNA Reporter on the Entire Well Surface or the Patterned Spot

The immobilization of both Cas12 and the ssDNA reporter on a 96-well ELISA plate surface was conducted as shown in Figure 3a. First, the carboxylic acid groups of a 96-well ELISA plate surface were activated by incubation with 200 mM EDC/NHS in 50 mM MES-NaOH buffer (pH 6.0) for 1 h. After the activation of the carboxylic acid groups, 250 nM ssDNA reporter and 12.5 nM NTA in 100 mM phosphate buffer (pH 7.5) were added dropwise on each spot and incubated for 12 h. The ssDNA reporter/NTA-immobilized surface was blocked by the incubation of 1 M ethanolamine hydrochloride in 100 mM phosphate buffer (pH 7.5) for 1 h. Subsequently, the Ni–NTA complex was formed by incubation with 100 mM nickel(II)chloride in water for 1 h. During incubation, crRNA was denatured at 95 °C for 5 min and then cooled from 95 to 4 °C at a rate of 0.1 °C/s. Finally, both 500 nM Cas12 and 1 μM crRNA in 2.5 μL of 1× NEBuffer 3 were immobilized on the surface by 2 h incubation. The surfaces were washed with water after each reagent immobilization step. The amount of reagent, amount of washing water, and number of washing steps for each immobilization step are listed in Table S4.

#### Patterning of the Dual-Target dsDNA Sensor

The spots for the dual-target sensor were patterned as shown in Figure S4. A microplate seal was cut into a circular shape (φ = 6.0 mm) with two holes (φ = 2 mm) using an auto-cutter. The cutting pattern was designed in Silhouette Studio (Silhouette, Lindon, UT, USA). Then, the seal was peeled from the backside film and adhered to the bottom surface of a 96-well ELISA plate modified with carboxylic acid groups.

#### Fluorescence Detection of dsDNA on the Cas12/ssDNA Reporter-Immobilized Surface

First, target dsDNA was dissolved in 1× NEBuffer 3 with 0.5 U/μL RNase Inhibitor. Then, the dsDNA solution (60 μL for the non-patterned well surface; 80 μL for the patterned well surface) was dropped into the wells and incubated at 37 °C for different periods. In the experiment to measure the cleavage ratio according to the reaction time (Figure 4) and the target dsDNA concentration (Figure 6), the reaction was stopped by incubation at 70 °C for 10 min for accurate evaluation under the short reaction and low target concentration conditions. After five rinses of the well with 300 μL of water, fluorescence images (λ_ex_ = 535–555 nm, λ_em_ = 570–625 nm) of the surface were obtained by microscopy and analyzed using Image J (NIH, Bethesda, MD, USA). The scale of fluorescence intensity was set as “0” for the surface without the ssDNA reporter and “1” for the surface-immobilized with [ssDNA reporter]/[NTA] = 250:12.5 nM when [target dsDNA] = 0 nM. The patterned well was filled with 1x NEBuffer 3 and covered with a polyolefin seal before the fluorescence measurements.

## Supporting Information Available

Data for the optimization of the ssDNA reporter concentration, fluorescence intensity data of the ssDNA-immobilized surface, Δ*F* values and cleavage ratios of SPCC, patterning process of 96-well, comparison of the workflow between the SPCC sensing model and DNA microarray, list of oligonucleotide sequences, comparison table of collateral cleavage-based multiplex nucleic acid detection, components of the PCR mixture, and the amount of reagents in immobilization steps (PDF)

## Abbreviations

CRISPR: clustered regularly interspaced short palindromic repeat
Cas12: CRISPR-associated protein 12
dsDNA: double-stranded DNA
crRNA: CRISPR RNA
SPCC: solid-phase collateral cleavage
ssDNA: single-stranded DNA
NAAT: nucleic acid amplification testing
PCR: polymerase chain reaction
LAMP: loop-mediated isothermal amplification
Cas9: CRISPR-associated protein 9
ELISA: enzyme-linked immunosorbent assay
F-Q reporter: ssDNA labeled with a fluorescent dye and quencher
CARMEN: combinatorial arrayed reactions for multiplexed evaluation of nucleic acids
Cas13: CRISPR-associated protein 13
HMP: hydrogel microparticle
HEX: hexachlorofluorescein
NTA: *N*_α_,*N*_α_-bis(carboxymethyl)-l-lysine hydrate
EDC: *N*-(3-dimethylaminopropyl)-*N*′-ethylcarbodiimide-hydrochloride
NHS: *N*-hydroxysuccinimide
PAM: protospacer adjacent motif
