# Supplementary material for: Solid-Phase Collateral Cleavage System Based on CRISPR/Cas12 and Its Application toward Facile One-Pot Multiplex Double-Stranded DNA Detection
Source: Bioconjug Chem. 2023 Oct 2;34(10):1754–65. doi: 10.1021/acs.bioconjchem.3c00294 (PMC10587867; doi:10.1021/acs.bioconjchem.3c00294)
Supplement: Supplementary file 1 — bc3c00294_si_001.pdf [file bc3c00294_si_001.pdf]

Supporting Information

Solid-Phase Collateral Cleavage System Based on CRISPR/Cas12 and Its Application Toward Facile One-Pot Multiplex Double-Stranded DNA Detection

Hiroki Shigemori[†,‡], Satoshi Fujita[†], Eiichi Tamiya[†,§], Shin-ichi Wakida[†,§], Hidenori Nagai[*,†,‡]

† Advanced Photonics and Biosensing Open Innovation Laboratory (PhotoBIO-OIL), National Institute of Advanced Industrial Science and Technology (AIST), Photonics Center Osaka University, 2-1 Yamada-Oka, Suita, Osaka 565-0871, Japan

‡ Graduate School of Human Development and Environment, Kobe University, 3-11 Tsurukabuto, Nada-ku, Kobe, Hyogo 657-0011, Japan

§ Institute of Scientific and Industrial Research (SANKEN), Osaka University, 8-1 Mihogaoka, Ibaraki, Osaka 567-0047. Japan

*Corresponding author:
Hidenori Nagai-†Advanced Photonics and Biosensing Open Innovation Laboratory (PhotoBIO-OIL), National Institute of Advanced Industrial Science and Technology (AIST), Photonics Center Osaka University, 2-1 Yamada-Oka, Suita, Osaka 565-0871, Japan; ‡Graduate School of Human Development and Environment, Kobe University, 3-11 Tsurukabuto, Nada-ku, Kobe, Hyogo 657-0011, Japan; 0000-0002-4663-7440
E-mail: hide.nagai@aist.go.jp

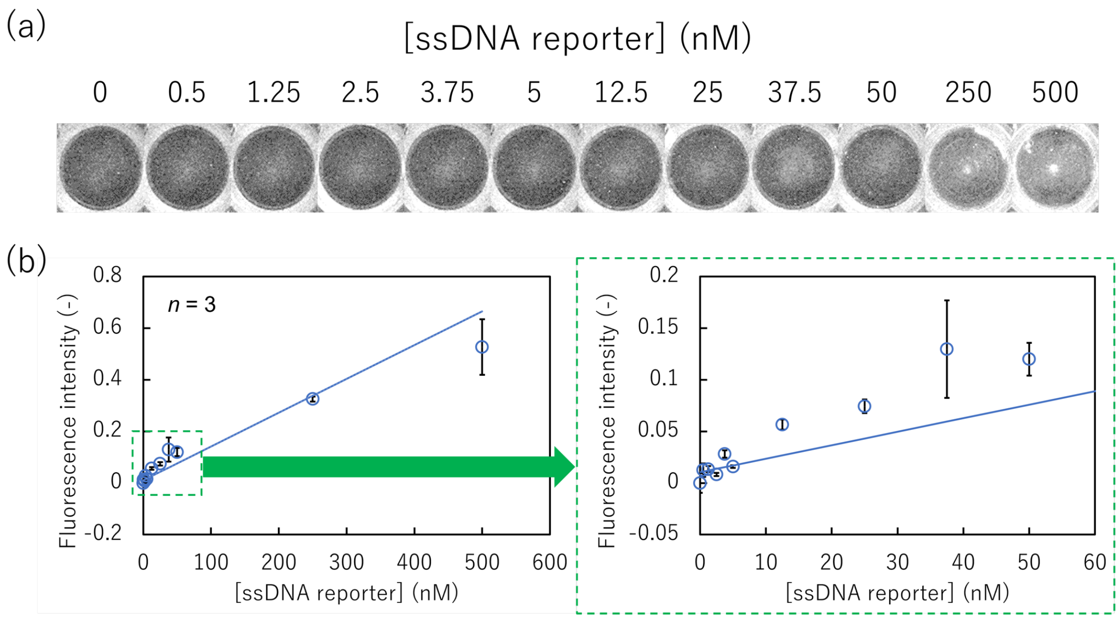

Figure S1. Optimization of the concentration of the ssDNA reporter for immobilization on the carboxylic acid groups of the well surface. (a) Fluorescence images of the well surface immobilized with various concentrations of ssDNA reporter (HEX-Poly A-NH_2-20nt). (b) Fluorescence intensity of the images (the blue line is the weighted calibration line fit to $y = 0.00131x + 0.01047$).

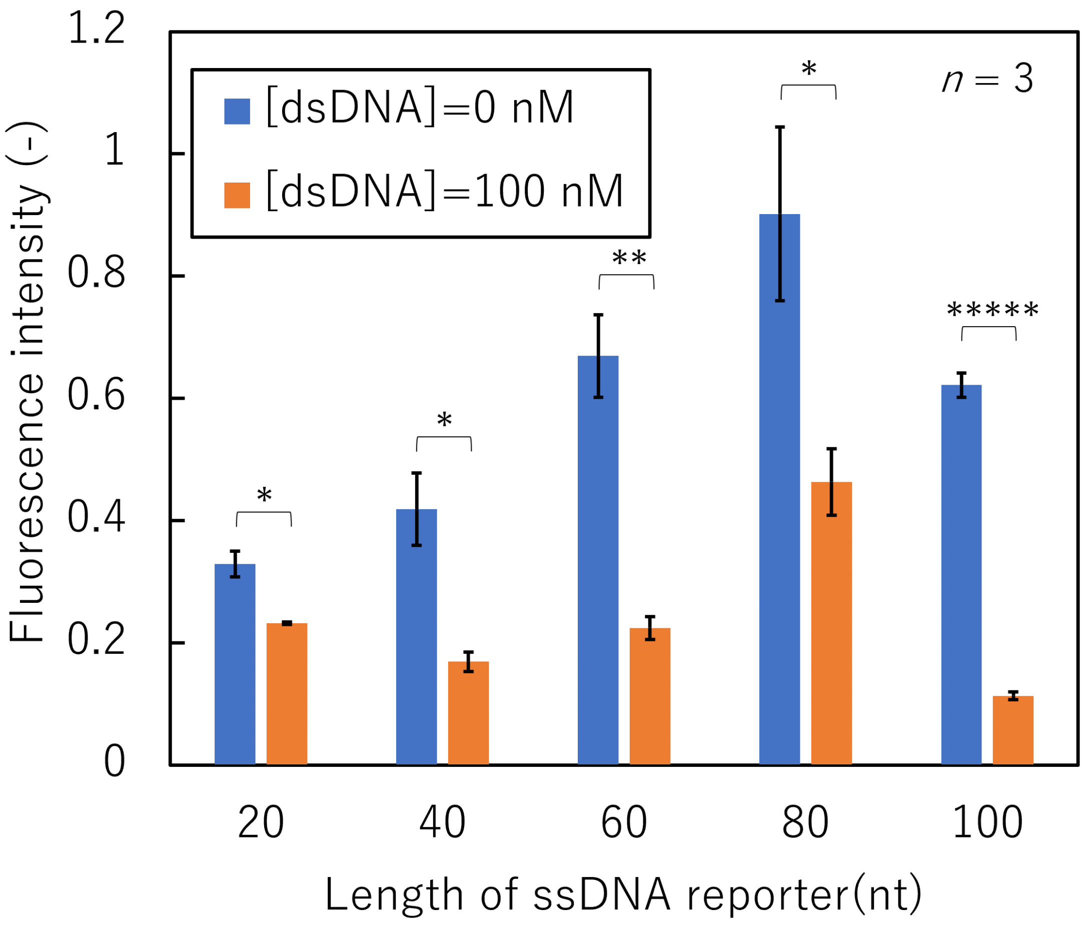

Figure S2. Fluorescence intensity of ssDNA reporter-immobilized surface in each reporter length condition (Two-tailed Student's t-test; n.s.: not significant, *: $p < 0.05$, **: $p < 0.01$, ***: $p < 0.005$, ****: $p < 0.001$, *****: $p < 0.0005$).

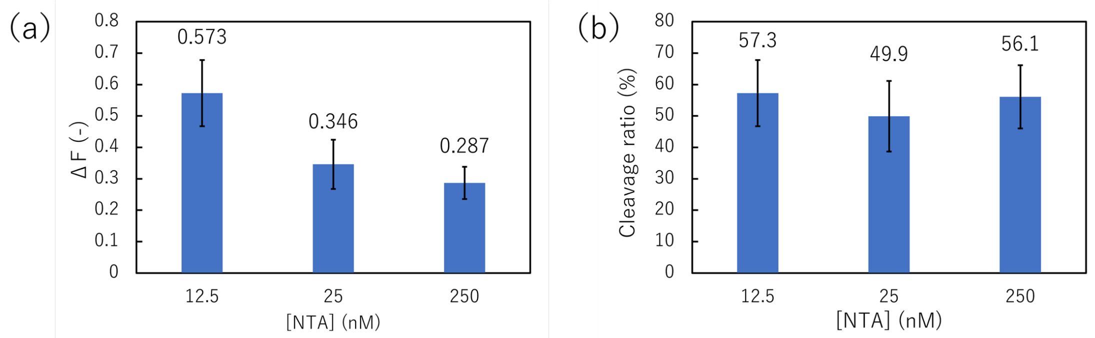

Figure S3. ΔF value (a) and cleavage ratio (b) of Cas12-crRNA/ssDNA reporter-immobilized surface in each [NTA] condition.

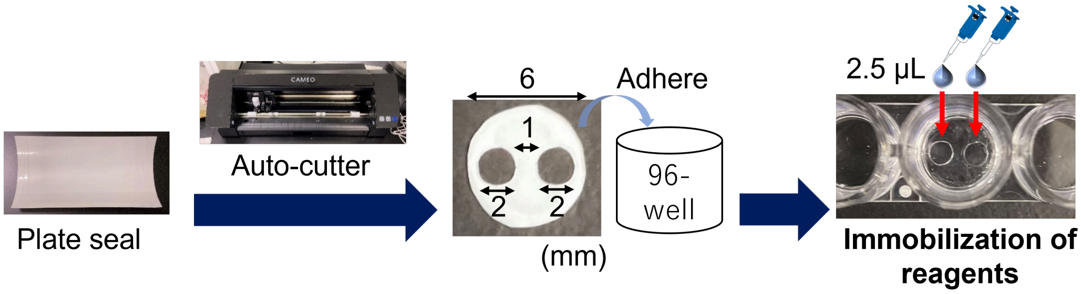

Figure S4. Fabrication of the dual-target dsDNA sensor on the bottom surface of the 96-well plate. (1) Cutting of the plate seal into a circular shape with two holes. (2) Adhesion of the cut seal to the bottom of the 96-well plate. (3) Dropping of 2.5 µL of each regent on each spot.

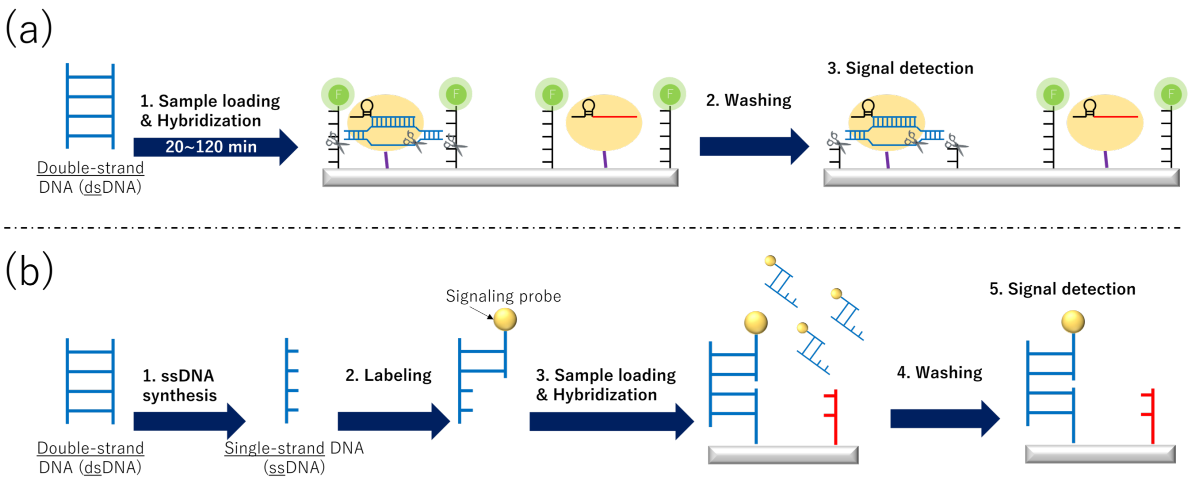

Figure S5. Comparison of workflow between (a) SPCC sensing model and (b) DNA microarray.[1]

Table S1. Oligonucleotide sequences of target dsDNA, primer, crRNA, and ssDNA reporter (the [ACC] sequence of target dsDNA amplicon was converted to TTT (part of the PAM sequence) by forward primer. Underline indicates the targeted region of the dsDNA and the target-recognizing region of the crRNA; Bold characters indicate mismatches between pEGFP-N1 and pVenus-N1).

**Amplified region of target dsDNA (sense strand)**	Sequence (5‘→3‘)
pEGFP-N1	CACCTACGGCAAGCTG[ACC]CTGAAG**T**T**C**ATCTGCACCACCGGCAAGCTGCCCGTGCCCTGGC<br>CCACCCTCGTGACCACCCTG**AC**CTACGGC**G**TGCAGTGCTTC**AG**CCGCTACCCCGACCACATG<br>AAGCAGCACGACTTCTTCAAGTCCGCCATGCCCGAAGGCTACGTCCAGGAGCGCACCATCTTCT<br>TCAAGGACG
pVenus-N1	CACCTACGGCAAGCTG[ACC]CTGAAG**C**T**G**ATCTGCACCACCGGCAAGCTGCCCGTGCCCTGGC<br>CCACCCTCGTGACCACCCTG**GG**CTACGGC**C**TGCAGTGCTTC**GC**CCGCTACCCCGACCACATG<br>AAGCAGCACGACTTCTTCAAGTCCGCCATGCCCGAAGGCTACGTCCAGGAGCGCACCATCTTCT<br>TCAAGGACG
Primer	
Forward	CACCTACGGCAAGCTG[TTT]CTGA
Reverse	CGTCCTTGAAGAAGATGGTGCGC
**crRNA**	
pEGFP-N1 targeting	UAAUUUCUACUAAGUGUAGAUUGAAG**U**U**C**AUCUGCACC
pVenus-N1 targeting	UAAUUUCUACUAAGUGUAGAUUGAAG**C**U**G**AUCUGCACC
**ssDNA reporter**	
HEX-Poly A-NH2-20nt	HEX-AAAAAAAAAAAAAAAAAAAA-AmC7
HEX-Poly T-NH2-20nt	HEX-TTTTTTTTTTTTTTTTTTTT-AmC7
HEX-Poly T-NH2-40nt	HEX-TTTTTTTTTTTTTTTTTTTTTTTTTTTTTTTTTTTTTTTT -AmC7
HEX-Poly T-NH2-60nt	HEX-TTTTTTTTTTTTTTTTTTTTTTTTTTTTTTTTTTTTTTTTTTTTTTTTTTTTTTTTTTTT -AmC7
HEX-Poly T-NH2-80nt	HEX-<br>TTTTTTTTTTTTTTTTTTTTTTTTTTTTTTTTTTTTTTTTTTTTTTTTTTTTTTTTTTTTTTTTTTTTTTTTT<br>TTTTTTT -AmC7
HEX-Poly T-NH2-100nt	HEX-<br>TTTTTTTTTTTTTTTTTTTTTTTTTTTTTTTTTTTTTTTTTTTTTTTTTTTTTTTTTTTTTTTTTTTTTTTTT<br>TTTTTTTTTTTTTTTTTTTTTTTTTT-AmC7

Table S2. Comparison of collateral cleavage-based multiplex nucleic acid detection

Categoly	Method	Targets	Assay spots	Chip size (cm^2)	Spots per Chip size ($/cm^2$)	Instruments	Operations	Ref.
**Microwell-based multi-chamber**	CARMEN	169	42400	32.15	1318.67	Droplet generator, corona treater and Fluorescent microscope	1. Droplet generation<br>2. Mixing emulsion<br>3. Sample inject<br>4. Color-coding<br>5. Merging droplets<br>6. Fluorescence detection	[2]
	mCARMEN	96	9216	109.14	84.44	Fluidic controller and fluorescence detector	1. Sample inject<br>2. Reagent inject<br>3. Running two instruments	[3]
**Microfluidic-based multi-chamber**	Finger-actuated microfluidic	8	8	27.84	0.29	Smartphone	1. Sample inject<br>2. Pushing button<br>3. Fluorescence detection	[4]
	Centrifugal microfluidic	2	32	54.10	0.59	Dedicated instrument for centrifuge and fluorescence detector	1. Sample inject<br>2. Running the instrument	[5]
	Radial PDMS microfluidic	9	30	14.06	2.13	Fluorescence microscope	1. Sample inject<br>2. Fluorescence detection	[6]
	μPADs	3	4	4.86	0.82	ChemiDoc™ MP imaging system	1. Sample inject<br>2. Fluorescence detection	[7]
**Combination of Cas12 and Cas13 subtypes**		4	1	N/A	N/A	Plate reader	1. Sample inject<br>2. Fluorescence detection	[8]
**Hydrogel microparticles (HMP)-based multi-chamber**		3	132	0.62	211.54	Fluorescence microscope	1. Mixing of sample and gel<br>2. Sample inject<br>3. Oil inject<br>4. Fluorescence detection	[9]
**SPCC-based sensor**		2	2	0.34	5.88	Fluorescence microscope	1. Sample inject<br>2. Washing<br>3. Fluorescence	This work

						detection	

Table S3. Components of PCR mixture. The reagents were added to tubes in order from top to bottom of the list. The 10x Fast Buffer I and dNTP mixture were part of the SpeedSTAR HS Polymerase kit.

Reagent	Concentration
**Nuclease-free water**	—
**10x Fast buffer I**	1x
**dNTP mixture (2.5 mM each)**	200 μM
**Forward primer**	0.4 μM
**Reverse primer**	0.4 μM
**SpeedSTAR HS Polymerase**	25 mU μL^{-1}
**pEGFP-N1 or pVenus-N1**	50 fg μL^{-1}

Table S4. Volumes of reagent and washing water and washing steps in each immobilization step.

Immobilization step	Single dsDNA detection		Dual-target dsDNA detection	
	Volume of reagent	Volume of washing water and no. of washing steps	Volume of reagent	Volume of washing water and no. of washing steps
EDC/NHS activation	100 μL	100 μL x 3	2.5 μL for each spot	4 μL x 3 for each spot
ssDNA & NTA immobilization	100 μL	100 μL x 1	2.5 μL for each spot	4 μL x 1 for each spot
Blocking	100 μL	100 μL x 1	2.5 μL for each spot	4 μL x 1 for each spot
Ni-NTA formation	100 μL	100 μL x 1	2.5 μL for each spot	4 μL x 2 for each spot
Cas12/crRNA immobilization	60 μL	100 μL x 5	2.5 μL for each spot	4 μL x 2 for each spot, followed by 300 μL x 3 for well

References

(1) Cleven, B. E. E.; Palka-Santini, M.; Gielen, J.; Meembor, S.; Krönke, M.; Krut, O. Identification and Characterization of Bacterial Pathogens Causing Bloodstream Infections by DNA Microarray. *Journal of Clinical Microbiology* **2006**, *44* (7), 2389–2397. DOI: 10.1128/jcm.02291-05.

(2) Ackerman, C. M.; Myhrvold, C.; Thakku, S. G.; Freije, C. A.; Metsky, H. C.; Yang, D. K.; Ye, S. H.; Boehm, C. K.; Kosoko-Thoroddsen, T.-S. F.; Kehe, J.; *et al.* Massively Multiplexed Nucleic Acid Detection with Cas13. *Nature* **2020**, *582* (7811), 277–282. DOI: 10.1038/s41586-020-2279-8.

(3) Welch, N. L.; Zhu, M.; Hua, C.; Weller, J.; Mirhashemi, M. E.; Nguyen, T. G.; Mantena, S.; Bauer, M. R.; Shaw, B. M.; Ackerman, C. M.; Thakku, S. G.; Tse, M. W.; *et al.* Multiplexed CRISPR-Based Microfluidic Platform for Clinical Testing of Respiratory Viruses and Identification of SARS-CoV-2 Variants. *Nat Med* **2022**, *28* (5), 1083–1094. DOI: 10.1038/s41591-022-01734-1.

(4) Xing, G.; Shang, Y.; Wang, X.; Lin, H.; Chen, S.; Pu, Q.; Lin, L. Multiplexed Detection of Foodborne Pathogens Using One-Pot CRISPR/Cas12a Combined with Recombinase Aided Amplification on a Finger-Actuated Microfluidic Biosensor. *Biosensors and*

Bioelectronics **2023**, *220*, 114885. DOI: 10.1016/j.bios.2022.114885.

(5) Chen, Y.; Mei, Y.; Jiang, X. Universal and High-Fidelity DNA Single Nucleotide Polymorphism Detection Based on a CRISPR/Cas12a Biochip. *Chem. Sci.* **2021**, *12* (12), 4455–4462. DOI: 10.1039/D0SC05717G.

(6) Xu, Z.; Chen, D.; Li, T.; Yan, J.; Zhu, J.; He, T.; Hu, R.; Li, Y.; Yang, Y.; Liu, M. Microfluidic Space Coding for Multiplexed Nucleic Acid Detection via CRISPR-Cas12a and Recombinase Polymerase Amplification. *Nat Commun* **2022**, *13* (1), 6480. DOI: 10.1038/s41467-022-34086-y.

(7) Yin, K.; Ding, X.; Li, Z.; Sfeir, M. M.; Ballesteros, E.; Liu, C. Autonomous Lab-on-Paper for Multiplexed, CRISPR-Based Diagnostics of SARS-CoV-2. *Lab Chip* **2021**, *21* (14), 2730–2737. DOI: 10.1039/D1LC00293G.

(8) Gootenberg, J. S.; Abudayyeh, O. O.; Kellner, M. J.; Joung, J.; Collins, J. J.; Zhang, F. Multiplexed and Portable Nucleic Acid Detection Platform with Cas13, Cas12a, and Csm6. *Science* **2018**, *360* (6387), 439–444. DOI: 10.1126/science.aaq0179.

(9) Roh, Y. H.; Lee, C. Y.; Lee, S.; Kim, H.; Ly, A.; Castro, C. M.; Cheon, J.; Lee, J.; Lee, H. CRISPR-Enhanced Hydrogel Microparticles for Multiplexed Detection of Nucleic Acids. *Advanced Science* **2023**, *10* (10), 2206872. DOI: 10.1002/advs.202206872.
